# Antimicrobial Resistance and Mortality in Carbapenem-Resistant *Pseudomonas aeruginosa* Infections in Southern Thailand

**DOI:** 10.3390/antibiotics14030322

**Published:** 2025-03-19

**Authors:** Parichart Chotimakorn, Sutthiporn Pattharachayakul, Yongyut Lertsrisatit, Wichai Santimaleeworagun, Pimpimon Tansakul, Mingkwan Yingkajorn, Sureerat Chelae, Rattanaruji Pomwised, Arnon Chukamnerd, Rosesathorn Soontarach, Sarunyou Chusri

**Affiliations:** 1 College of Pharmacotherapy Thailand, Nonthaburi 11000, Thailand; pchotimakorn@gmail.com; 2 Department of Pharmacy, Bhumibol Adulyadej Hospital, Bangkok 10220, Thailand; 3 Department of Clinical Pharmacy, Faculty of Pharmaceutical Sciences, Prince of Songkla University, Songkhla 90110, Thailand; sutthiporn@pharmacy.psu.ac.th (S.P.); yongyut@pharmacy.psu.ac.th (Y.L.); 4 Department of Pharmacy, Faculty of Pharmacy, Silpakorn University, Nakorn Pathom 73000, Thailand; swichai1234@gmail.com; 5 Department of Pharmacognosy and Pharmaceutical Botany, Faculty of Pharmaceutical Sciences, Prince of Songkla University, Songkhla 90110, Thailand; pimpimon.t@psu.ac.th; 6 Department of Pathology, Faculty of Medicine, Prince of Songkla University, Songkhla 90110, Thailand; mingkwan.psu@gmail.com (M.Y.); sureerath.j@psu.ac.th (S.C.); 7 Division of Biological Science, Faculty of Science, Prince of Songkla University, Songkhla 90110, Thailand; rattanaruji.p@psu.ac.th; 8 Division of Infectious Diseases, Department of Internal Medicine, Faculty of Medicine, Prince of Songkla University, Songkhla 90110, Thailand; arnonchukamnerd@hotmail.com (A.C.); rosesathron_pim@hotmail.com (R.S.)

**Keywords:** carbapenem-resistant *Pseudomonas aeruginosa*, efflux pump, porin, β-lactamase, mortality, risk factors

## Abstract

**Background/Objectives**: Carbapenem-resistant *Pseudomonas aeruginosa* (CRPA) is an important pathogen associated with high mortality and treatment failure rates. We aimed to assess the susceptibility of CRPA to antipseudomonal agents, identify its resistance mechanisms, and evaluate clinical outcomes in a sample of CRPA isolates. **Methods**: This was an in vitro study of a clinical isolate of CRPA from hospitalized patients with CRPA infection and a retrospective observational study of these patients, who were diagnosed between 14 February 2021 and 10 August 2023 at Songklanagarind Hospital in Songkhla, Thailand. In vitro experiments were conducted to determine the minimum inhibitory concentrations (MICs) of the antipseudomonal agents using the broth microdilution method. Resistance mechanisms were assessed using the modified carbapenem inactivation method, combined disk tests, and quantitative real-time reverse transcription polymerase chain reaction. **Results**: A total of 140 CRPA isolates were analyzed. Both traditional and novel β-lactams had high MICs. The most common resistance mechanism was the upregulation of the MexAB-OprM efflux pump (81.3%), followed by the downregulation of the OprD porin (48.9%) and metallo-β-lactamase (MBL) production (45.0%), and the overexpression of *bla*_AmpC_ (41.0%). The 30-day all-cause mortality rate was 30.5%. The risk factors associated with 30-day mortality included a Charlson Comorbidity Index of ≥5 (OR: 3.43; 95% CI: 1.07–10.99; *p* = 0.03), sepsis (OR: 10.62; 95% CI: 1.26–89.44; *p* = 0.03), and septic shock (OR: 4.39; 95% CI: 1.67–11.55; *p* < 0.01). In contrast, receiving active documented therapy was significantly associated with reduced mortality (OR: 0.17; 95% CI: 0.04–0.74; *p* = 0.01). **Conclusions**: This study revealed higher MIC values of all β-lactams for CRPA, while colistin and amikacin remained effective. The resistance mechanisms included MexAB-OprM overexpression, OprD downregulation, MBL production, and *bla*_AmpC_ overexpression, with a higher prevalence of MBL than in other regions of Thailand. High 30-day mortality was associated with comorbidities, sepsis, and septic shock, but active therapy reduced mortality.

## 1. Introduction

*Pseudomonas aeruginosa* is a major cause of severe nosocomial infections worldwide. In 2005, a previous study reported it as the most commonly isolated pathogen (18.1%) in nosocomial pneumonia and the second most common (16.3%) in urinary tract infections in the United States [1]. It is associated with severe outcomes, including a 40–60% mortality rate and a 42% treatment failure rate [2,3]. Moreover, it is intrinsically resistant to multiple classes of antimicrobial agents, and the emergence of multidrug-resistant (MDR) strains poses a growing challenge in many countries. The limited availability of other effective treatments has increased the reliance on carbapenems and also contributed to the growing prevalence of carbapenem-resistant *P. aeruginosa* (CRPA) [4,5,6,7]. Clinical outcome data obtained from 206 hospitals in the U.S. between 2009 and 2013 indicate that CRPA infections increase the risk of in-hospital mortality 1.2-fold, are associated with a high incidence of treatment failure, and result in significantly longer hospital stays and higher treatment costs compared with carbapenem-susceptible *P. aeruginosa* infections [4,8]. According to a study by Li et al. (2014), the overall 30-day mortality rate for CRPA infection was 27.0% [9], while Dantas et al. (2018) found a higher rate of 47.3% in the carbapenem-resistant group [10], and, in 2017, the World Health Organization identified it as one of the top three critical pathogens that require urgent research into new antimicrobial agents [11]. The U.S. Centers for Disease Control and Prevention (CDC) reported an incidence of 14.3% for CRPA infections in 2019 [12]. In Thailand, National Antimicrobial Resistance Surveillance Thailand data show an increasing trend of *P. aeruginosa* infections from 2000 to 2021, with resistance against imipenem rising from 10.7% to 22.1% and resistance against meropenem increasing from 17.8% in 2015 to 19.3% in 2021 [13]. Notably, *P. aeruginosa* has the second highest rate of imipenem resistance in Thailand, with the southern region reporting a CRPA incidence of 22.7% [14].

CRPA resistance to carbapenems involves three main mechanisms. The first is the increased activity of efflux pumps, particularly the MexA, MexB, and OprM (MexAB-OprM) pump [15,16], which significantly enhances resistance to certain carbapenems (a 32-fold increase in the minimal inhibitory concentration [MIC] of meropenem and an 8-fold increase in that of doripenem). The second is decreased drug penetration due to the downregulation of the OprD porin on the outer membrane; this has been reported to result in high-level resistance to imipenem (MIC > 32 mg/L) and intermediate resistance to meropenem [17]; this mechanism has been associated with a 2.65-fold increase in mortality [18]. The third mechanism involves the production of carbapenemases, particularly class B metallo-β-lactamases (MBLs) such as imipenemase (IMP) and Verona Integron-encoded MBL (VIM) [19]. This can considerably complicate treatment, and IMP-producing CRPA has been associated with a 5-fold increase in the risk of mortality [20]. These enzymes can hydrolyze nearly all β-lactams, leading to elevated MICs that exceed the effective levels of antimicrobials. Additionally, although class C or AmpC β-lactamases typically do not affect carbapenem, exposure to β-lactams can induce the overexpression of *bla*_AmpC_ by 100–1000 times, particularly when combined with the downregulation of *oprD*, which may further increase the MICs of meropenem and doripenem by 2–4 times [17]. Several factors contribute to mortality in patients with CRPA infection, including age, severity score, sepsis or septic shock, inappropriate empirical therapy, a delay in receiving the appropriate antimicrobial treatment, hospital-acquired infections, and the source of the infection [8,9,21,22,23,24,25,26].

Local in vitro data on the prevalence of resistance mechanisms, antimicrobial susceptibility testing (AST), and MIC distributions for CRPA are essential for optimal antimicrobial selection and dosing, as inappropriate or delayed treatment can increase mortality [26]. In cases where CRPA remains susceptible to traditional β-lactams (e.g., aztreonam, ceftazidime, cefepime, piperacillin–tazobactam), these agents may still be effective. However, if resistance to these agents is present but susceptibility to novel β-lactam/β-lactamase inhibitors (e.g., ceftazidime–avibactam and ceftolozane–tazobactam) remains, these novel agents may serve as alternative treatment options. Additionally, clinical outcome data are crucial for identifying risk factors for mortality and improving patient care. However, there is currently a lack of detailed data regarding the resistance mechanisms, AST, MIC distributions and clinical outcomes of CRPA infections in Thailand. This single-center study aimed to evaluate the AST and MIC data of traditional and novel antipseudomonal β-lactams, investigate the prevalence of resistance mechanisms in clinical CRPA isolates, and assess 30-day all-cause mortality and its associated risk factors in patients with CRPA infection.

## 2. Results

### 2.1. In Vitro Analysis

#### 2.1.1. Antimicrobial Susceptibility Testing

A total of 140 CRPA clinical isolates were obtained from participants diagnosed with and treated for CRPA infections. Notably, 133 of these isolates (95%) were classified as healthcare-associated. The majority of the isolates were obtained from sputum samples (88 isolates, 62.9%), followed by urine (22 isolates, 15.7%) and ascitic fluid samples (9 isolates, 6.4%). Other sources included other tissues, blood, abscess fluid, bile, and bronchoalveolar lavage. The susceptibility of these 140 isolates to various antimicrobial agents was evaluated through MIC analysis, and the results indicated that 124 isolates (88.6%) were MDR. The susceptibility percentages, MIC ranges, and MIC50 and MIC90 values are provided in Table 1.

#### 2.1.2. Evaluation of Resistance Mechanisms

**Figure 1:**
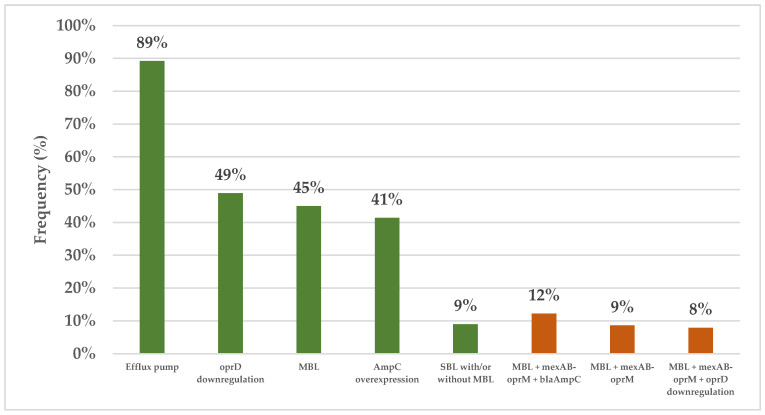
The prevalence of different resistance mechanisms in the CRPA isolates. The green and orange bars represent the frequencies of the individual resistance mechanisms and multiple distinct resistance mechanisms, respectively. Efflux pumps: At least one of *mexAB-oprM*, *mexCD-oprJ*, *mexEF-oprN*, or and *mexXY-oprM*. Abbreviations: CRPA, carbapenem-resistant *Pseudomonas aeruginosa*; MBL, metallo β-lactamase; SBL, serine β-lactamase.

All 140 clinical isolates were evaluated for carbapenemase production using the modified carbapenem inactivation method (mCIM). The results showed that 75 isolates (53.6%) were positive for carbapenemase, 63 (45.0%) were negative, and 2 (1.4%) were indeterminate. Subsequently, the combined disk test (CDT) was used to assess MBL production in the carbapenemase-positive isolates, with the results revealing that 63 isolates (45.0%) produced MBL and 12 isolates (8.6%) produced serine β-lactamase (SBL) with or without MBL. All the clinical isolates also underwent genotypic testing using quantitative real-time reverse transcription polymerase chain reaction (qRT–PCR), but one isolate was excluded due to its cDNA concentration being lower than required. The results for the 139 remaining isolates revealed significant insights into their resistance mechanisms. Of the 139 isolates, 124 (89.2%) exhibited the upregulation of at least one efflux pump, with *mexAB-oprM* being the most common (upregulation in 113 samples, 81.3%), followed by *mexXY-oprM* (in 43 isolates, 30.7%), *mexEF-oprN* (in 33 isolates, 23.7%), and *mexCD-oprJ* (in 8 isolates, 5.8%). Additionally, 68 isolates (48.9%) exhibited the downregulation of *oprD*, while 57 isolates (41.4%) exhibited the upregulation of *bla*_AmpC_. Notably, 127 isolates (90.7%) were found to possess multiple resistance mechanisms, with 56 isolates (40.3%) having three mechanisms and 43 isolates (30.9% having two mechanisms, whereas 21 isolates (15.1%) had a single mechanism. The most common combination involved MBL production alongside *mexAB-oprM* and *bla*_AmpC_ upregulation (in 17 samples, 12.23%), followed by MBL production with *mexAB-oprM* upregulation (in 12 samples, 8.63%) and MBL production with *mexAB-oprM* upregulation combined with the downregulation of *oprD* (in 11 samples, 7.91%) (Figure 1).

The MIC50 values of all the β-lactam antimicrobial agents analyzed, except for aztreonam, increased at least 2-fold in the MBL-producing CRPA strains compared to the non-producing strains. For the non-β-lactam antimicrobial agents, no significant differences were observed between these two groups (Table 2).

### 2.2. Clinical Data

The majority of the included patients were male (97 patients, 69.3%), 105 (75%) were aged 60 years or older (median age: 72 years, IQR: 59–81), and 121 (86.4%) had a Charlson Comorbidity Index (CCI) of ≥3 (median CCI: 6; IQR: 4–7). A total of 61 patients (43.6%) were admitted to the intensive care unit, and 45 patients (32.1%) presented with septic shock. The severity of illness was assessed using Acute Physiology and Chronic Health Evaluation II (APACHE II) scores, yielding a median score of 16 (IQR: 12–21), while the median Sequential Organ Failure Assessment (SOFA) score was 4 (IQR 2–7). A total of 124 patients (88.6%) were infected with MDR CRPA, 85 (60.7%) with extensively drug-resistant (XDR) CRPA, and 92 (65.7%) with difficult-to-treat resistant (DTR) CRPA. The most common infection site was the lungs (90 patients, 64.3%), followed by the urinary tract (26 patients, 18.6%). Additionally, CRPA was detected in the bloodstream of 9 patients (6.4%), and 78 patients (55.7%) had polymicrobial infections. A total of 94 patients (68.1%) received active empirical therapy, while 31 patients (22.5%) received active documented therapy. Most patients (103, 73.6%) were treated with monotherapy, predominantly with colistin (68 patients, 48.6%). Acute kidney injury occurred in 23.3% of patients post-treatment, particularly among those who received colistin.

#### 2.2.1. The 30-Day All-Cause Mortality Rate

Among the 140 non-duplicated CRPA isolates obtained from 140 patients, 30-day mortality data could not be tracked for 12 (8.6%) patients, as they were lost to follow-up, leaving 128 patients for analysis. In these patients, 39 patients died within 30 days (30-day mortality rate: 30.5%). Additionally, 23 patients died within 14 days (14-day mortality rate, 17.2%), with data unavailable for 6 patients (4.3%). Furthermore, 15 patients died within 7 days (7-day mortality rate, 10.9%), with data unavailable for 2 patients (1.4%). Treatment failure was observed in 53 patients (37.9% of the cohort). The characteristics of the 128 patients included in the 30-day mortality analysis are summarized in Table 3.

#### 2.2.2. Factors Associated with 30-Day All-Cause Mortality

The univariate analysis of the factors affecting the 30-day mortality rate revealed significant associations with several variables, including a CCI ≥5, the presence of invasive medical devices, an APACHE II score ≥15, a SOFA score ≥2, septic shock, lung infection, MBL-producing strain infections, co-infection with *Stenotrophomonas maltophilia*, receiving inactive empirical therapy, and receiving inactive documented therapy. The multiple logistic regression analysis results showed that a CCI ≥ 5 (OR, 3.43; 95% CI, 1.07–10.99; *p* = 0.03), SOFA score ≥ 2 (OR, 10.62; 95% CI, 1.26–89.44; *p* = 0.03), and septic shock (OR, 4.39; 95% CI, 1.67–11.55; *p* < 0.01) significantly increased the risk of 30-day mortality. Conversely, receiving active documented therapy (OR, 0.17; 95% CI, 0.04–0.74; *p* = 0.01) was associated with a significant reduction in the 30-day mortality rate (Table 4).

## 3. Discussion

Infections caused by CRPA are a growing global health concern and are characterized by their high resistance to antimicrobials of multiple classes, complicating treatment and contributing to elevated morbidity and mortality rates. This study aimed to assess the in vitro activities of CRPA isolates with antipseudomonal agents, identify prevalent resistance mechanisms, and examine clinical outcomes, including 30-day all-cause mortality and related factors, in patients with CRPA infections admitted to Songklanagarind Hospital.

For the 140 CRPA isolates analyzed, the MICs of colistin and amikacin were low, with MIC50 values of ≤1 mg/L and ≤ 8 mg/L, respectively, consistent with previous studies [25,28,29,30,31,32,33]. However, a notable resistance to β-lactams was observed, with novel agents such as ceftazidime–avibactam and ceftolozane–tazobactam showing susceptibility rates of 32.9% and 27.9%, respectively, and high MIC values (MIC50, 256 mg/L). These results contrast with those of several studies from Europe and the U.S., where higher susceptibility rates to these agents were reported [34,35,36,37,38,39,40]. Our findings suggest that the prevalence of MBL production could explain the high resistance to β-lactams. This prevalence was higher than those reported in other studies, including those from Thailand, with that of the *bla*_VIM_ gene being the most common, followed by *bla*_IMP_ [34,41,42]. In contrast, the *bla*_KPC_, *bla*_GES_, or *bla*_OXA-48_ genes encoding SBLs were found to be relatively rare in our study, consistent with previous reports [42]. However, there may be instances where both SBLs and MBLs are co-expressed, leading to false negative results in phenotypic testing, which uses ethylenediaminetetraacetic acid (EDTA) to inhibit MBL activity, but not SBLs [43]. To date, there have been no reports in Thailand of the co-expression of SBLs and MBLs [41,42]. The non-MBL-producing strains exhibited lower MICs and higher susceptibilities to all β-lactams compared to the MBL-producing strains, consistent with other studies [25]. However, their susceptibility remained relatively low (<60%) due to the presence of other resistance mechanisms, such as MexAB-OprM efflux pump and *bla*_AmpC_ upregulation. These mechanisms were highly prevalent in the strains in this study, contributing to their reduced susceptibility to novel agents like ceftazidime–avibactam and ceftolozane–tazobactam [44]. We also observed a lower prevalence of *oprD* downregulation compared to other studies [34,41], likely due to the high prevalence of MBL production, *bla*_AmpC_ upregulation, and efflux pump activity, which can reduce the need for *oprD* downregulation. Additionally, excluding strains from the same patient at different time points may have underestimated this mechanism. Our study found that 20.7% of CRPA isolates were susceptible to piperacillin–tazobactam and 22.9% were susceptible to ceftazidime. Additionally, 28.6% of isolates were susceptible to at least one of these antimicrobials. Nearly all of these isolates were susceptible to novel agents such as ceftazidime–avibactam and ceftolozane–tazobactam (susceptibility rates of 97.9% and 93.6%, respectively). These isolates were all non-MBL-producing strains, with efflux pump upregulation being the most prevalent mechanism (85.0%), primarily involving MexAB-OprM (72.5%), followed by *oprD* downregulation (75.0%). In contrast, *bla*_AmpC_ upregulation was relatively rare (15.0%). The co-occurrence of MexAB-OprM upregulation and *oprD* downregulation was the most common co-mechanism (50.0%). In non-MBL-producing strains resistant to ceftazidime–avibactam or ceftolozane–tazobactam, we found that efflux pump overexpression was the most prevalent (94.4%), primarily involving MexAB-OprM (88.9%). The other mechanisms identified included *oprD* downregulation (44.4%), *bla*_AmpC_ overexpression (41.7%), and SBLs (33.3%). The most prevalent co-mechanism was the simultaneous overexpression of *mexAB-oprM* and *bla*_AmpC_ (38.9%). The overexpression of *mexXY-oprM* is often associated with resistance to ciprofloxacin and gentamicin, while *mexEF-oprN* and *mexCD-oprJ* are primarily linked to resistance to fluoroquinolones [16,17,41,45,46]. In this study, *mexEF-oprN*, *mexXY-oprM*, and *mexCD-oprJ* were found at low frequencies, but 80% of the total studied isolates showed resistance to ciprofloxacin. This resistance likely results from mechanisms beyond the efflux pumps tested in this study, such as mutations in the quinolone-resistance-determining region of the DNA gyrase (*gyrA*) and topoisomerase IV (*parC*) genes [47,48]. Regarding aminoglycosides, the prevalence of the *mexXY-oprM* mechanism (30.7%) was similar to the resistance rate to gentamicin (32.1%). However, amikacin showed a very low resistance (4%), possibly due to the influence of other untested mechanisms that affect gentamicin more than amikacin, such as acetylate (AAC(3′)-I, III, IV, V) or adenylate (ANT(2″)) aminoglycoside-modifying enzymes.

The 30-day all-cause mortality rates in our study were similar to those reported by Buehrle et al. in their retrospective cohort study on CRPA bacteremia [8]. However, our mortality rates were higher than those observed by Li et al., likely due to the absence of carbapenemase-producing CRPA strains in their study [9]. In two studies conducted in Thailand, the mortality rates due to MDR Gram-negative bacteria and XDR-PA infections were similar to those in our study, which may be attributed to the similarities in the characteristics of the patient populations (majority over 60 years old and having multiple comorbidities, as well as infections primarily affecting the lower respiratory tract and urinary tract). However, neither of these studies included a subgroup analysis specifically related to the outcomes of CRPA infections [49,50]. Multiple logistic regression analysis revealed that a CCI ≥ 5, SOFA score ≥ 2, and septic shock were significant risk factors for 30-day mortality while active documented therapy reduced mortality, and these findings align with those of several international studies [9,21,22,49,51]. Our study did not find that MBL production significantly affected mortality in logistic regression analysis, which contrasts with the findings of other studies [20]. However, we found that MBL production was more prevalent in patients who died, which could be attributed to the relatively small sample size, potentially limiting the statistical power to detect a meaningful relationship.

Our results also diverge from those of several studies supporting the use of novel agents in the empirical treatment of CRPA infections. However, the studies investigating ceftolozane–tazobactam involved small sample sizes of CRPA isolates with low MICs [52,53,54], and the studies on ceftazidime–avibactam primarily focused on ceftazidime-resistant strains, not carbapenem-resistant strains like CRPA [55,56,57,58]. Although imipenem–cilastatin–relebactam has demonstrated efficacy in treating imipenem-resistant *P. aeruginosa*, the exclusion of MBL-producing strains limits its applicability in regions with high MBL prevalence [59]. Aztreonam–avibactam has shown potential in restoring aztreonam activity against MBL-producing carbapenem-resistant Enterobacterales by preventing degradation via β-lactamases producing Enterobacterales and AmpC β-lactamases, but its efficacy against CRPA remains uncertain [60]. Some in vitro studies have reported a lack of synergy against MBL-producing CRPA, possibly due to additional resistance mechanisms such as efflux pumps and OprD porin downregulation [61]. Further research is needed to optimize the clinical application of these agents in CRPA infections. Cefiderocol, preferred for MBL-producing infections [62,63,64], has demonstrated efficacy in severe carbapenem-resistant Gram-negative infections [65]; however, recent reports suggest its reduced effectiveness due to mutations in AmpC- and TonB-dependent receptors [62,65,66].

Understanding local CRPA AST is crucial to guide empiric antimicrobial therapy while awaiting AST results. The high prevalence of MBLs and *bla*_AmpC_ overexpression in healthcare-associated CRPA infections remains a challenge. Our findings highlight the consideration of empiric β-lactams therapy, including with novel agents. However, their use should be reserved for documented therapy when susceptibility to these agents is confirmed, in alignment with the ESCMID and IDSA guidance [62,63]. Non-carbapenemase-producing CRPA strains that remain susceptible to traditional β-lactams (i.e., aztreonam, ceftazidime, cefepime, piperacillin–tazobactam) may be effectively treated with these agents, making them preferable to carbapenems in carbapenem-sparing strategies. Notably, this approach has not been significantly associated with increased mortality [67]. Given the time-dependent bactericidal activity of β-lactams, several clinical data and Monte Carlo simulation studies support their use, based on optimized dosing regimens, extended infusion strategies, and MIC values, to enhance efficacy and improve clinical outcomes [68]. Cefepime (2 g every 8 h over 4 h) achieved a ≥90% probability of target attainment (PTA) for 60% fT ≥ MIC at an MIC ≥ 8 mg/L [68]. Piperacillin/tazobactam (4.5 g every 6 h over 3 h) attained a ≥90% PTA for 50% fT ≥ MIC at an MIC of 16/4 mg/L and 60% at 32/4 mg/L [69]. High-dose extended infusion meropenem (2 g every 8 h over 3 h) may be used when MIC values are ≤16 mg/L, while continuous infusions (4–8 g/day) achieved a ≥90% PTA for 100% fT ≥ MIC at an MIC ≤ 8 mg/L in critically ill patients [70]. The combination of extended meropenem infusion with fosfomycin (16 g continuous infusion) provided a PTA of approximately 80%, whereas 24 g/day of fosfomycin prolonged infusion for 6 h achieved a >90% PTA for non-MDR CRPA, despite MIC90 values of >32 mg/L for meropenem and >1024 mg/L for fosfomycin [71]. Non-MBL-producing CRPA isolates that are non-susceptible to traditional β-lactam agents but remain susceptible to novel agents (i.e., ceftolozane–tazobactam, ceftazidime–avibactam, and imipenem–cilastatin–relebactam), should be treated with these agents, particularly in infections outside the urinary tract, where alternative options may be limited [72]. MBL-producing CRPA isolates are generally resistant to all β-lactam agents and often exhibit high MIC values. Cefiderocol is recommended for such infections [72]; however, clinical data on it are limited, and it remains unavailable in Thailand. Consequently, treatment may require the use of non-β-lactam agents such as aminoglycosides or colistin, which remain effective against CRPA with low MIC values. However, these agents should be reserved as last-resort options, particularly for infections outside the urinary tract, due to lower clinical cure rates and higher nephrotoxicity [73]. Additionally, their clinical utility is limited by poor epithelial fluid penetration. Despite these challenges, local resistance patterns may necessitate their use in empiric treatment to ensure adequate CRPA coverage. For CRPA resistant to all novel agents, combination therapy is recommended, especially for severe infections, with additional agents selected based on MIC values [62,63,64,74].

Our study has several limitations. First, the healthcare-associated nature of our CRPA isolates may have led to an overestimation of certain resistance mechanisms and MIC values. Second, excluding isolates from the same patient to avoid duplicates may have resulted in an underestimation of some resistance mechanisms. Third, the lack of genotyping for carbapenemase enzymes, testing for other resistance mechanisms not covered in this study, and the absence of MIC data for novel agents such as cefiderocol, limits the depth of our analysis. Fourth, the retrospective design of our study introduces inherent limitations, including potential selection bias due to the inclusion of only patients with clinical isolates obtained per hospital protocol, leading to possible case exclusion. Additionally, recall bias may have arisen from missing or incomplete medical records, potentially affecting data accuracy and comprehensiveness. Finally, the small sample size constrained a more comprehensive investigation, and the presence of polymicrobial infections in most patients may have influenced the results. While our study provides valuable insights, it does not define an optimal regimen for reducing CRPA-related mortality.

## 4. Materials and Methods

### 4.1. Study Design and Participants

This was an in vitro study of a clinical isolate of CRPA from hospitalized patients with CRPA infection and retrospective observational study of these patients, who were diagnosed between 14 February 2021 and 10 August 2023 at Songklanagarind Hospital in Songkhla, Thailand. This hospital is a tertiary medical center in southern Thailand and reported the second-highest incidence of CRPA infections in 2019. We included all adult patients aged 18 years or more who received a first diagnosis of CRPA infection according to the CDC/NHSN surveillance definitions for specific infection types [75]. Patients who had not received treatment for their CRPA infections were excluded from the study. In vitro testing was performed to assess antimicrobial susceptibility, determine the corresponding MICs, and evaluate the possible resistance mechanisms.

### 4.2. Definitions

We defined a CRPA isolate as resistant (R) if it exhibited resistance to at least one carbapenem with antipseudomonal properties (imipenem, meropenem, or doripenem). Carbapenem resistance was determined based on a MIC ≥ 8 mg/L, as assessed using the broth microdilution method [43]. MDR *P. aeruginosa* was defined as a strain that was non-susceptible to at least one agent in three or more antimicrobial classes. XDR *P. aeruginosa* was defined as a strain that was non-susceptible to at least one agent in all but two or fewer antimicrobial classes. DTR *P. aeruginosa* was defined as a strain that was non-susceptible to all antipseudomonal antimicrobials, including piperacillin–tazobactam, ceftazidime, cefepime, aztreonam, meropenem, imipenem–cilastatin, ciprofloxacin, and levofloxacin [62,64,76]. A healthcare-associated infection was classified as an inpatient case with 3 or more days of hospitalization or a patient with a history of previous hospitalization, surgery, or long-term care facility residence in the past year. Additionally, it was also classified in cases with the insertion of an indwelling device in the previous 7 days or chronic dialysis at the assessment time. None of these risk factors were used to classify a community-associated infection [30]. We defined the clinical outcome as all-cause mortality occurring within 30 days from the date of diagnosis of CRPA infection. Treatment failure was defined as clinical treatment failure meeting any of the following conditions: the persistence or progression of all signs and symptoms after 72 h of therapy; the development of new clinical findings consistent with active infection; and death due to infection [77,78,79]. CCI is a clinical tool that assigns weighted scores to comorbidities to assess disease burden and predict mortality risk. We calculated the CCI using underlying conditions abstracted from the medical records. APACHE II score is a scoring system used in intensive care units (ICUs) to assess disease severity and predict patient mortality based on physiological variables, age, and chronic health conditions. Sepsis was defined as a SOFA score of 2 or more points. Septic shock was defined as life-threatening organ dysfunction, serum lactate levels >2 mmol/L, and persisting hypotension despite adequate volume resuscitation, requiring a vasopressor to maintain mean arterial pressure > 65 mmHg [80]. Active empirical therapy was defined as at least 1 antimicrobial agent with in vitro activity within 24 h. Active documented therapy was defined as at least 1 antimicrobial agent with in vitro activity after obtaining the AST results [81].

### 4.3. Clinical Data Collection

The medical records of patients were reviewed to obtain data on demographics and the clinical outcomes of infection, including age, gender, hospital ward, underlying diseases, source of infections, critically ill status, immunocompromised status, the duration of hospitalization, the use of invasive medical devices, antimicrobial therapy, and clinical laboratory test results.

### 4.4. Sample Collection and Isolate Identification

All non-duplicated CRPA isolates were obtained from the leftover specimens of patients admitted to the hospital due to CRPA infection between 14 February 2021 and 10 August 2023. The bacterial samples were stored in 20% glycerol at −80 °C for further in vitro experiments. *P. aeruginosa* identification was performed using biochemical methods and confirmed by matrix-assisted laser desorption/ionization time-of-flight mass spectrometry at the hospital’s clinical laboratory. *P. aeruginosa* were restreaked on tryptic soy agar (TSA) plates and the plates were incubated at 37 °C for 18–24 h. The bacterial stock was prepared by inoculating a single colony into tryptic soy broth (TSB) tubes and incubating at 37 °C for 4–6 h accordingly. Then, a glycerol was added into the culture tubes and they were stored at −80 °C for further in vitro experiments.

### 4.5. AST and MIC Determination

The AST and MIC determination for all the CRPA clinical isolates was performed using the broth microdilution method for ceftolozane–tazobactam, ceftazidime–avibactam, piperacillin–tazobactam, ceftazidime, cefepime, aztreonam, meropenem, imipenem, and doripenem. Additionally, ciprofloxacin, colistin, amikacin, and gentamicin were assessed using an automated broth microdilution system (Sensititre^TM^ Vizion^TM^; Thermo Fisher Scientific, Waltham, MA, USA). *P. aeruginosa* ATCC 27853 was used as the quality control strain. Bacterial growth was monitored using 0.015% resazurin as an indicator. All experiments were performed in triplicate. Susceptibility and MIC breakpoints were interpreted according to CLSI 2023 guidelines, except for of gentamicin, for which CLSI 2022 standards were used [27].

### 4.6. Phenotypic Detection of Carbapenemase Production

The carbapenemase production by all the clinical isolates was assessed using the mCIM, following the protocol and conducting interpretation according to the CLSI guidelines [43]. For isolates testing positive via the mCIM, the CDT was performed to further assess MBL production, as previously described [82]. In brief, the isolate was incubated in TSB for 4–5 h and then adjusted to a 0.5 McFarland standard using 0.85% NaCl. The suspension was inoculated onto an MHA plate, onto which a ceftazidime disk (30 µg) and a ceftazidime disk supplemented with 8 µL of 50 mM EDTA were placed. The plate was incubated at 37 °C for 16–18 h. After incubation, the zones of inhibition were measured. An increase in clear zone diameter of ≥8 mm indicated a positive result for MBL production.

### 4.7. Genotypic Detection of Efflux Pump, Porin, and bla_AmpC_ Genes

qRT–PCR was used to assess the levels of transcription of specific resistance-related genes in all isolates. The sequences of the validated primers for the target genes *mexB*, *mexD*, *mexF*, *mexY*, *oprD*, *bla*_AmpC_, and the housekeeping gene *rpsL* are provided in Table 5. Nucleotide sequences were retrieved from the GenBank database, and primers were designed using the Primer-BLAST tool. The selected primers proved effective when tested under optimized PCR conditions. Total RNA was extracted from the isolates in the late log phase using the PureLink^®^ RNA Mini Kit (Invitrogen^TM^; Thermo Fisher Scientific, Waltham, MA, USA). The extracted total RNAs were evaluated for quality and quantity using agarose gel electrophoresis and spectrophotometry. Following this assessment, the RNAs were treated with RNase-free DNase I (Invitrogen^TM^) to remove any contaminating DNA, enabling us to synthesize their first-strand cDNA by reverse transcription using the Invitrogen^TM^ SuperScript^TM^ VILO^TM^ cDNA Synthesis Kit (Invitrogen^TM^; Thermo Fisher Scientific, Waltham, MA, USA). Finally, the concentration of the cDNA template was measured using spectrophotometry at an OD_260_ (Nanodrop lite, Thermo Fisher Scientific, Wilmington, DE, USA). The qualified cDNA sample was kept at −20 °C until used.

The qRT-PCR was performed using the PowerUp^TM^ SYBR^TM^ Green Master Mix (Applied Biosystems^TM^, Thermo Fisher Scientific, Wilmington, DE, USA) and the QuantStudio^®^ 3 Real-Time PCR Instrument (Thermo Fisher Scientific, Wilmington, DE, USA). The cDNA was diluted to a final concentration of 10 ng for the reactions. The reactions were performed for 2 min at 95 °C, followed by 40 cycles of 15 s at 95 °C and 60 s at 60 °C. A dissociation curve analysis was performed at the end. Amplifications were performed in duplicates for each gene, and the average Ct values from two experiments (standard deviation < 0.1) were normalized against a reference housekeeping gene. Expression levels were presented as fold change ratios compared to *P. aeruginosa* PAO1.

The target genes *mexD*, *mexF*, *mexY*, and *bla*_AmpC_ were classified as upregulated if their expression ratios were at least 10-fold higher than those in *P. aeruginosa* PAO1. The gene *mexB* was classified as downregulated when its ratio was at least 3-fold greater. In contrast, *oprD* was considered downregulated if its expression was more than 40% lower than in *P. aeruginosa* PAO1 [17,41,83].

### 4.8. Statistical Analysis

SPSS version 18 (IBM Corp., Armonk, NY, USA) was used to analyze all data in this study. Continuous variables were compared using Student’s *t*-test or the Mann–Whitney U test. Categorical variables were compared using the chi-square or Fisher’s exact test. Multivariate logistic regression analysis was used to identify prognostic factors associated with mortality among those with a *p*-value < 0.05 in the univariate analysis. A *p*-value < 0.05 was considered statistically significant.

## 5. Conclusions

The results of this study showed the MIC values of novel β-lactams for the analyzed CRPA isolates were high, but that those of colistin and amikacin remained low. The main resistance mechanisms included the upregulation of the MexAB-OprM efflux pump, downregulation of the OprD porin, MBL production, and *bla*_AmpC_ overexpression, with a surprisingly higher prevalence of MBL production than reported in other regions of Thailand. This study found a high 30-day mortality rate among patients with CRPA infections and identified several key risk factors. Septic shock was a significant predictor of poor outcomes, and multiple comorbidities further increased mortality risk. However, active documented therapy significantly reduced mortality, emphasizing the importance of appropriate and timely treatment.

## Figures and Tables

**Table 1:** Antimicrobial susceptibility of CRPA isolates (*n* = 140).

Antimicrobial Agent	S (%)	I (%)	R (%)	MIC Range (mg/L)	MIC50 (mg/L)	MIC90 (mg/L)
Ceftazidime–avibactam	32.86	NA	67.14	≤0.5–≥512	256	≥512
Ceftolozane–tazobactam	27.86	0.00	72.14	≤0.5–≥512	256	≥512
Piperacillin–tazobactam	20.71	10.71	68.57	2–512	128	256
Cefepime	20.71	6.43	72.86	≤1–≥1024	256	≥1024
Ceftazidime	22.86	2.14	75.00	≤1–≥1024	≥1024	≥1024
Aztreonam	14.29	51.43	34.29	2–≥128	16	≥128
Doripenem	20.00	5.71	74.29	≤0.5–≥512	128	256
Meropenem	7.14	5.71	87.14	1–≥512	128	≥512
Imipenem	2.86	7.14	90.00	1–≥512	64	256
Ciprofloxacin	15.00	5.00	80.00	0.06–≥2	≥2	≥2
Colistin	NA	98.60	1.40	≤1–4	≤1	2
Amikacin	89.30	6.40	4.30	≤8–≥64	8	32
Gentamicin	66.40	1.40	32.10	2–>8	2	>8

The MICs were determined using broth microdilution, except for those for ciprofloxacin, colistin, amikacin, and gentamicin, which were determined using an automated micro-broth dilution testing system (Sensititre^TM^ Vizion^TM^ system; Thermo Fisher Scientific, Waltham, MA, USA). The AST and MIC breakpoints are those according to the Clinical and Laboratory Standards Institute (CLSI) 2023 (except for those for gentamicin, which are according to the CLSI 2022) [27]. Abbreviations: S, susceptibility; I, intermediate; R, resistance; NA, not available; MIC, minimal inhibitory concentration; MIC50, minimal inhibitory concentration that inhibited 50% of the isolates; MIC90, minimal inhibitory concentration that inhibited 90% of the isolates.

**Table 2:** Comparison of MIC values and susceptibilities of MBL-producing and non-MBL-producing CRPA isolates to individual antimicrobial agents.

Antimicrobial Agents	MBL-Producing Strains		Non-MBL-Producing Strains	
	MIC50 (mg/L)	S (%)	MIC50 (mg/L)	S (%)
Ceftazidime–avibactam	≥512	0	4	59.70
Ceftolozane–tazobactam	≥512	0	4	50.60
Piperacillin–tazobactam	128	0	32	37.70
Cefepime	512	0	32	37.70
Ceftazidime	≥1024	0	64	41.60
Aztreonam	16	4.80	16	22.10
Doripenem	256	0	8	36.40
Meropenem	≥512	0	16	13.00
Imipenem	128	0	8	5.20
Ciprofloxacin	≥2	0	≥2	27.30
Colistin	≤1	NA	≤1	NA
Amikacin	≤8	100.00	≤8	80.50
Gentamicin	≤2	65.00	≤2	67.50

**Table 3:** General baseline characteristics of patients with CRPA infection (*n* = 128).

Characteristics	Survived (*n* = 89)	Died (*n* = 39)
Male, *n* (%)	62 (69.7)	27 (69.2)
Age, median (IQR), year	71 (55–82)	69 (63–80)
Body mass index (BMI), median (IQR), kg/m^2^	21.4 (18.1–24.0)	19.9 (18.7–22.8)
Serum creatinine, median (IQR), mg/dL	0.7 (0.5–0.9)	0.8 (0.5–1.5)
eGFR, median (IQR), mL/min/1.73 m^2^	93.0 (77.0–109.0)	83.5 (35.0–122.5)
Comorbidities		
Cancer	30 (33.7)	14 (35.9)
Diabetes	21 (23.6)	14 (35.9)
Asthma or chronic obstructive pulmonary disease	22 (24.7)	10 (25.6)
Multiple comorbidities (>1)	42 (47.2)	29 (74.4)
Charlson Comorbidity Index (CCI), median (IQR)	5 (4–7)	6 (5–8)
Invasive medical device insertion, *n* (%)	61 (68.5)	34 (87.2)
Immunosuppressive therapy within 14 days *, *n* (%)	17 (19.1)	7 (17.9)
Neutropenia, *n* (%)	5 (5.6)	2 (5.1)
APACHE II score, median (IQR)	14 (11–19)	20 (16–25)
SOFA score, median (IQR)	3 (1–5)	8 (5–10)
Septic shock, *n* (%)	14 (15.7)	24 (61.5)
Intensive care unit admission, *n* (%)	31 (34.8)	25 (64.1)
Site of infection		
Lower respiratory tract	51 (57.3)	33 (84.6)
Urinary tract	20 (22.5)	3 (7.7)
Intra-abdominal	7 (7.9)	1 (2.6)
Skin and soft tissues	6 (6.7)	1 (2.6)
Bloodstream	4 (4.5)	1 (2.6)
Bone and joints	1 (1.1)	-
Secondary septicemia, *n* (%)	5 (5.6)	3 (7.7)
Inadequate source control, *n* (%)	6 (6.7)	1 (2.6)
Polymicrobial infection (>1 bacterial species) ^a^, *n* (%)	48 (53.9)	23 (59.0)
Resistance mechanism of CRPA		
Overexpression of efflux pumps	80 (89.9)	35 (89.7)
Downregulation of *oprD* porin	39 (43.8)	21 (53.8)
MBL production	34 (38.2)	23 (59.0)
*bla*_AmpC_ overexpression	34 (38.2)	17 (43.6)
Active empirical therapy, *n* (%)	64 (71.9)	22 (56.4)
Active documented therapy, *n* (%)	25 (28.1)	3 (7.7)
Monotherapy of documented therapy, *n* (%)	70 (78.7)	27 (69.2)
Colistin	20 (22.5)	18 (46.2)
β-lactams	26 (29.2)	7 (17.9)
Fluoroquinolones	13 (14.6)	1 (2.6)
Aminoglycosides	10 (11.2)	1 (2.6)
Fosfomycin	1 (1.1)	-
Combination of documented therapy, *n* (%)	19 (21.3)	12 (30.8)
Colistin combination-based therapy	14 (15.7)	10 (25.6)
Colistin with β-lactams	7 (7.9)	6 (15.4)
Colistin with fluoroquinolones	3 (3.4)	2 (5.1)
Colistin with fosfomycin	4 (4.5)	2 (5.1)
Non-colistin combination-based therapy	5 (5.6)	2 (5.1)
Adverse drug reaction		
Acute kidney injury (AKI) ^¶^	17 (19.1)	11 (31.4)
Treatment failure or clinical failure, *n* (%)	12 (13.5)	34 (87.2)

* Chemotherapy, immunosuppressants, or corticosteroids (equivalent to ≥20 mg of prednisolone for at least 2 weeks). ^a^ refers to organisms isolated together from the same specimen. ^¶^ indicates serum a creatinine increase ≥0.3 mg/dL within 48 h, or ≥1.5 times the baseline, or a urine output of less than 0.5 mL/kg/h over 6 h. Abbreviations: IQR, interquartile range; eGFR, estimated glomerular filtration rate; APACHE II, Acute Physiology and Chronic Health Evaluation II; SOFA, Sequential Organ Failure Assessment.

**Table 4:** Factors associated with 30-day mortality in patients with CRPA infection (*n* = 128).

Variables	Univariate Analysis		Multivariate Analysis	
	OR (95%CI)	*p* Value	OR (95%CI)	*p* Value
Age > 60 years	1.97 (0.80–4.81)	0.13		
CCI ≥ 5	4.40 (1.57–12.35)	<0.01 *	3.43 (1.07–10.99)	0.03 **
Invasive device insertion	3.12 (1.10–8.83)	0.02 *		
APACHE II score ≥ 15	5.35 (2.13–13.40)	<0.01 *		
SOFA score ≥ 2	17.44 (2.27–133.52)	<0.01 *	10.62 (1.26–89.44)	0.03 **
Septic shock	8.57 (3.62–20.28)	<0.01 *	4.39 (1.67–11.55)	<0.01 **
ICU admission	3.34 (1.52–7.33)	<0.01 *		
Lower respiratory tract infection	4.09 (1.56–10.76)	<0.01 *		
Urinary tract infection	0.28 (0.08–1.03)	0.04 *		
MBL production	2.32 (1.07–5.01)	0.03 *	2.39 (0.91–6.23)	0.07
*S. maltophilia* co-infection	3.05 (0.87–10.69)	0.08 *		
Active empirical therapy	0.46 (0.21–1.02)	0.05 *		
Active documented therapy	0.20 (0.05–0.73)	<0.01 *	0.17 (0.04–0.74)	0.01 **

* *p* < 0.1. ** *p* < 0.05. Abbreviations: CCI, Charlson Comorbidity Index; ICU, intensive care unit; MBL, metallo β-lactamase.

**Table 5:** Primers used in this study.

Gene	Primer	Sequence (5′->3′)	Product Length (bp)
*mexB*	mexB_F	CCACCCACGGCTACGAG	200
	mexB_R	CACCTGGGTACGCTCGG	
*mexD*	mexD_F	CAGACCGCTACCCTGGTG	152
	mexD_R	ACCAGGACCATCGCTTCG	
*mexF*	mexF_F	TCCCGGCTCGAACGC	127
	mexF_R	GGAGCCGCGGACGAA	
*mexY*	mexY_F	GGTGGACGACGCGATCA	199
	mexY_R	CGCGAACTGGCGGTAGA	
*oprD*	oprD_F	AACATCTACCGCACAAACGAT	160
	oprD_R	GGCCGAAGCCGATATAATCAA	
*bla*_AmpC_	AmpC_F	ACAGATCCGCGACTACTACC	152
	AmpC_R	GGAACACTTGCTGCTCCAT	
*rpsL*	rpsL_F	AACTCGGCACTGCGTAA	194
	rpsL_R	CGGTCTTTGACACCCGA	

Abbreviations: bp, base pairs.

## Data Availability

The required data have been included in this article. For additional information, please contact the corresponding authors.
